# A high-resolution species-level dataset of global marine ornamental imports into the United Kingdom

**DOI:** 10.1038/s41597-026-07852-6

**Published:** 2026-07-17

**Authors:** Joanna M. Murray, Sarah R. Alewijnse, Olivia Harrod, David Hughes, Isla MacMillan, Christopher Popham, Emily Roebuck

**Affiliations:** 1 Centre for Environment, Fisheries and Aquaculture Science (Cefas), Pakefield Road, Lowestoft, Suffolk NR33 0HT UK; 2 Centre for Environment, Fisheries and Aquaculture Science (Cefas), Barrack Road, Weymouth, DT4 8UB UK

## Abstract

The global marine aquarium trade moves thousands of fish and invertebrate species, mostly from Indo-Pacific coral reefs to aquariums in the United States of America, Europe, and East Asia. Characterising which species are traded, in what volumes, and from where remains difficult due to complex supply chains, with shipment import records providing the most reliable data. This dataset presents species-level information on marine ornamental imports into the UK during selected periods in 2018 and 2019. Sales invoices from London Heathrow Airport’s Border Control Post were collected, scanned, digitised, and standardised. In total, 287 invoices containing marine species were processed, documenting 1,664 taxa, 82% identified to species level across 241 families and 20 classes. Non-scleractinian invertebrates accounted for the highest trade volumes, while bony fishes represented most species traded and over half of the trade’s value. This high-resolution dataset provides new insight into the UK’s role in the global marine aquarium trade and supports efforts to identify vulnerable species and promote more sustainable and transparent trade practices.

## Background & Summary

Trade in marine wildlife spans a diverse array of species and products ranging from commercially harvested species for seafood like sharks, rays, and sea cucumbers, to dried seahorses for Traditional Chinese Medicine, and live reef fish and stony corals which supply the international aquarium trade^1–5^.

Currently there are around 2,000 marine species listed under the Convention on International Trade in Endangered Flora and Fauna (CITES) most of which are in Appendix II. Listing in Appendix II means that trade in these species is allowed but must be regulated to ensure it is legal, sustainable, and traceable. Many more species, such as marine ornamental fishes and non-scleractinian invertebrates, are not listed at all, so their trade is legal by default. Reporting of trade in these species is often informal and declared using broad commodity codes such as ‘Live ornamental fish (030110)’ and ‘Live ornamental saltwater fish (03011090)’. Trade reporting at this resolution limits our understanding about which species are traded and in what volumes, making the identification of vulnerable populations to international trade problematic^6^.

Whilst most marine ornamental fish harvested for the aquarium trade are not listed within CITES, their conservation management and trade have been on the CITES agenda since the European Union, Switzerland and United States of America submitted CoP18 Doc. 94, recommending a workshop to examine the conservation implications of the trade. A summary of the workshop prepared by the CITES Secretariat can be found in a AC33 Doc. 4 (Rev. 2). Since the workshop in May 2024, the Animals Committee provided draft decisions for consideration at CoP20 (Nov 2025) which includes an invitation to Parties, to where possible, implement recording of international trade data on marine ornamental fishes at the species level^7^.

Species-level data on traded ornamental fish and invertebrates in the UK have been sought using wholesaler records^8^ and mystery shopper surveys of retailers^9^, but globally, best available information has been derived from import records^10–13^. Sales notes or invoices associated with imported consignments fully document trade from the exporting country, providing quantities and the value of each species.

To better understand the UK’s trade in marine ornamental species in terms of diversity, volume, value and origin, and support the provision of data to the CITES Marine ornamental Fishes Technical Workshop in May 2024, we sampled, scanned and digitised consignment documentation including health certificates, CITES permits where required, and sales invoices. Species-level data from sales invoices were cleaned and standardised to provide a high-resolution snapshot of marine ornamental imports into the UK. More broadly, these data contribute to a growing body of information about trade in non-CITES listed marine ornamental fish which can aid the identification of species warranting further research or management intervention.

## Methods

### Consignment documents

Live ornamental fish imported into the UK using the commodity codes ‘Live ornamental fish (030110)’ and ‘Live ornamental saltwater fish (03011090)’ are required to have a valid animal health certificate demonstrating that animal health requirements have been met, and a CITES permit if importing endangered species listed under the Convention’s appendices. A packing list or sales invoice which provides species-level information on the quantity and value of species within the consignment is also provided within the documentation. Consignments must enter the UK through a border control post (BCP) which is designated to check live ornamental fish.

Hard copies of imported consignment documents were obtained from the Animal and Plant Health Agency (APHA) at Heathrow Animal Reception Centre (HARC), London Heathrow Airport BCP, under a data sharing agreement. It is estimated that around 70% of ornamental fish arriving by air into the UK arrive at London Heathrow Airport BCP (pers comm, City of London Corporation at Heathrow Animal Reception Centre).

Given the volume of documents, it was not feasible to process all documents held, so a sample was taken which included all imports from the second week of selected months from March 2018 to November 2019. Although the sampled months were chosen to represent different periods throughout the year, the dataset does not include all months and therefore does not provide full annual coverage. This may limit its use for assessing monthly variation in traded species.

Consignment documents were manually scanned and digitised in Microsoft Excel. Along with species, digitisers recorded the number of individuals, the value (unit and total cost), the aquatic environment declared on the invoice (marine, freshwater or both), and whether the organisms were declared to be wild caught or captive bred, as detailed in the sales invoice. Only marine species are provided in this dataset, and all commercially sensitive information has been redacted. It should be noted that during the data collation period, the UK was still part of the European Union and imports were handled using the Trade and Control Expert System (TRACES).

### Data standardisation

**Fig. 1:**
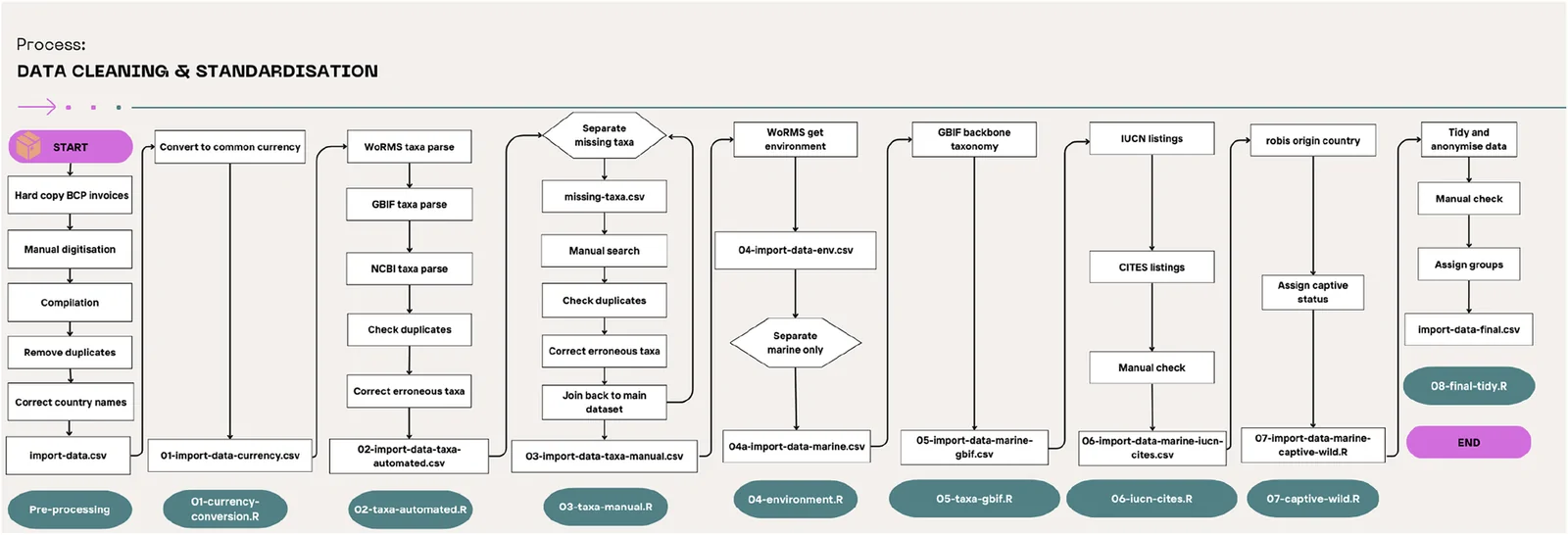
Schematic of the data cleaning and standardisation process.

Once all sales invoices containing marine species from the sample had been digitised, data were cleaned to standardise records using R version 4.5.1^14^ (Table 1, Fig. 1). First, all currencies were converted to USD using a single static exchange rate (XE Currency, 10 September 2019). Although exchange rates may have fluctuated during the study period, these variations were assumed to be small and unlikely to affect the study’s macroeconomic conclusions. Next, we used the World Register of Marine Species^15^, Global Biodiversity Information Facility^16^, and National Center for Biotechnology Information^17^ to correct species names, and acquire taxonomic information (Family, Order and Class). We used the worrms^18^, rgbif^19^ and taxize^20^ packages to interface with the respective databases. Multiple databases were used to best capture the plethora of taxa names used in invoices. In cases where only common names were recorded, or where incorrect names were not detected as synonyms, we manually identified the taxa. Taxa were also manually corrected where misspellings were not detected by the code. We also used WoRMS to identify the environment in which the species lives: marine, freshwater or brackish and those assigned to freshwater and brackish were excluded from further analyses.

**Table 1:** Fields and associated descriptions for standardised import data.

Field	Description
UID	Unique ID for entry
Binomial	Binomial scientific name according to GBIF backbone taxonomy
Group	Taxonomic grouping. Either bony fishes, other vertebrates, stony corals, other invertebrates, or algae
Class	Taxonomic class according to GBIF backbone taxonomy
Order	Taxonomic order according to GBIF backbone taxonomy
Family	Taxonomic family according to GBIF backbone taxonomy
Genus	Taxonomic genus according to GBIF backbone taxonomy
Species	Taxonomic species according to GBIF backbone taxonomy. Left blank where entry is only identified to genus level.
Rank	Taxonomic rank to which the entry is identified, according to GBIF backbone taxonomy.
Origin_country	Origin country for the entry. Countries named according to ISO 3166
Import_date	Date of import into the UK, in ISO 8601 format
Number	Number of individuals in the entry
Unit_cost	Cost per individual
Total_cost	Total cost of the entry
Currency	Currency for Unit_cost and Total_cost, as per the original invoice. Currency codes according to ISO 4217
Value_USD	Total cost of the entry in United States dollars. Conversions taken from XE currency on 2019-09-10
IUCN_listing	Conservation listing according to IUCN assessments. • LC = Least Concern • NT = Near Threatened • VU = Vulnerable • EN = Endangered • DD = Data Deficient • NA = no assessment
CITES_listing	CITES listing according to Species + database. • II = CITES Appendix II • NC = Taxon removed from appendices
Captive_wild	Captive bred or wild caught status. • wild_declared = Declared wild caught • farmed_declared = Declared captive bred • farmed_likely = Likely farmed based on range and origin country • NA = Captive bred or wild caught status unknown
AWB_ID	Pseudonymised invoice number

Next, we standardised all taxonomy to the GBIF backbone taxonomy^21^. Given the large number of non-fish taxa in our dataset, we prioritised using databases with broad taxonomic coverage and found that the GBIF backbone integrates well with subsequent data cleaning and standardisation steps, including the IUCN Red List API, while also closely aligning with the taxonomies used by IUCN and CITES. We used IUCN and CITES listings to ascertain the conservation status of the species in the record. IUCN listings were acquired using the rredlist package^22^, which interfaces with the IUCN Red List API. We downloaded CITES listings from the Species + website^23^.

Where source (wild caught or cultivated) was declared, records were labelled as “Declared wild” or “Declared farmed”. Where species’ origin countries were outside of their range, these records were labelled as “Likely farmed”. We ascertained species’ ranges using the robis package^24^, which interfaces with the Ocean Biodiversity Information System^25^ API. Finally, we selected relevant columns (Table 1) and pseudonymised the invoice number.

## Data Records

Species-level information was derived from 287 sales invoices which were sampled during select months in 2018 and 2019, using the methods described above. There are 32,861 entries including 1,664 different taxa, 1,372 (82%) of which were identified to species level, across 241 families and 20 classes. Non-scleractinian invertebrates comprise the largest share of the dataset by volume, while bony fishes represented the majority of species traded and over half the trade’s value. The dataset can be accessed online via the Cefas Data Portal^26^.

## Technical Validation

Taxonomic names were compiled using the most reliable information available at the time of sourcing to ensure entries are accurate; however, we were reliant on the accuracy of species identifications reported by exporters on sales invoices, and therefore some misidentifications may be present in the dataset. Any erroneous entries should be flagged to the corresponding author. All technical validation was conducted in R version 4.5.1^14^.

To assess how representative the dataset is of total marine ornamental species imported into the UK, we used a species accumulation curve where we treated each sales invoice as a site. Within each invoice, we considered only those records where the taxon was identified to species level. We used the specaccum function from the vegan package^27^ to calculate the species accumulation, using random permutation and polygonal confidence intervals.

We estimated the “true” number of species (*S*_*true*_) imported using the Chao estimator for species richness data:

$${S}_{t{\rm{rue}}}={S}_{o{\rm{bs}}}+\frac{{F}_{1}^{2}}{2{F}_{2}}$$

where *S*_*obs*_ is the observed number of species, *F*_*1*_ is the number of species which occur only once, and *F*_*2*_ is the number of species which occur exactly twice.

**Fig. 2:**
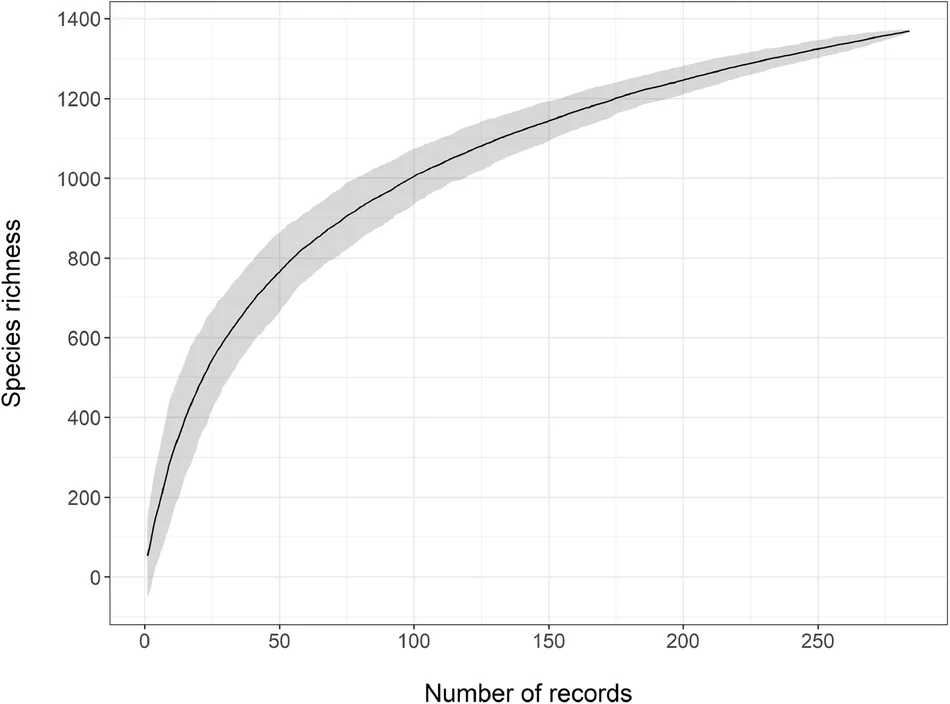
Species accumulation curve of marine species imported into the UK sampled during 2018 and 2019. The grey shading indicates the standard deviation of species richness permutations.

The species accumulation curve indicates that the dataset does not encapsulate the full diversity of species imported into the UK (Fig. 2). The Chao estimator for the total species pool was 1,715 ± 49. While some species were imported in large volumes, some species were imported only a handful of times, with 91 species only appearing in a single invoice during the sampled period.

## Usage Notes

UK trade data from HM Revenue and Customs can be accessed online to obtain the value (£) and Net Mass (kg) of imports into the UK, including those into Heathrow in 2018 and 2019. UK trade data does not provide species-level information but could be used alongside our data to understand the frequency of imports through Heathrow BCP compared to other ports of entry.

## Data Availability

Code used to prepare and analyse the data for this paper are available at https://github.com/CefasRepRes/uk-marine-ornamentals. The code is available for use under the Open Government Licence (OGL) v3: http://www.nationalarchives.gov.uk/doc/open-government-licence/version/3. Please note, some sensitive information in the code has been redacted for this public version. The code was run using R version 4.5.1.
